# Comparison of Various Vagal Maneuvers for Supraventricular Tachycardia by Network Meta-Analysis

**DOI:** 10.3389/fmed.2021.769437

**Published:** 2022-02-03

**Authors:** Edward Pei-Chuan Huang, Chi-Hsin Chen, Cheng-Yi Fan, Chih-Wei Sung, Pei Chun Lai, Yen Ta Huang

**Affiliations:** ^1^Department of Emergency Medicine, National Taiwan University Hospital Hsin-Chu Branch, Hsinchu City, Taiwan; ^2^Department of Emergency Medicine, National Taiwan University Medical College and Hospital, Taipei City, Taiwan; ^3^Education Center, College of Medicine, National Cheng Kung University Hospital, National Cheng Kung University, Tainan, Taiwan; ^4^Department of Surgery, College of Medicine, National Cheng Kung University Hospital, National Cheng Kung University, Tainan, Taiwan

**Keywords:** vagal maneuver, supraventricular tachycardia, standard Valsalva maneuver, modified Valsalva maneuver, carotid sinus massage, network meta-analysis

## Abstract

**Background:**

Vagal maneuvers (VagMs) are recommended as the first-line treatment of supraventricular tachycardia (SVT). However, the optimal type of VagMs remains unproven.

**Aim:**

This study aims to compare the effectiveness and adverse events amongst VagMs on SVT *via* network meta-analyses (NMAs).

**Methods:**

We systematically searched randomized controlled trials (RCTs) that involved adults with SVT and compared VagMs without language restrictions. We determined the initial and final responses of conversion rate to sinus rhythm and adverse events. Risk of bias (RoB) was appraised by Cochrane revised tool, and contribution matrix was calculated. NMAs were synthesized using frequentist random-effects model and presented as relative risk (RR) with 95% CI. The order of probability was presented as surface under the cumulative ranking curve analysis (SUCRA). Sensitivity analysis was performed using both Bayesian and frequentist approach with fixed- or random-effects models. Certainty of evidence (CoE) was rated by using the Grading of Recommendations, Assessment, Development, and Evaluations methodology.

**Results:**

Fourteen RCTs with 2,180 patients were enrolled. Small portion of mixed estimates was contributed from high overall RoB studies. Compared with carotid sinus massage (CSM), the modified Valsalva maneuver (MVM) was the most effective VagM after initial performance [SUCRA: 0.9992, RR: 5.47 (1.77–16.93)] and at the end of study [SUCRA: 1.0000, RR: 3.62 (2.04–6.39), CoE: high]. The standard VM did not elicit better conversion rate to the sinus rhythm than CSM at the initial response [SUCRA: 0.4395, RR: 1.97 (0.63–6.15)] and at the end of the study [SUCRA: 0.4795, RR: 1.64 (0.94–2.87), CoE: moderate]. The SUCRA value of CSM at the initial and final responses was the least one amongst three VagMs (0.0613 and 0.0205, respectively). Adverse events amongst three VagMs were similar (CoE: low). Sensitivity analyses yielded consistent results.

**Conclusion:**

We recommended MVM as the first choice of VagM for rhythm conversion before the pharmacological management of SVT.

## Introduction

Supraventricular tachycardia (SVT) or paroxysmal SVT is a cardiogenic emergency. Annually, its incidence is ~1.8 per 10,000 visits to the emergency department (ED) in the United States of America (1). SVT may lead to palpitations, chest pain, or dyspnea. SVT may further contribute to hemodynamic instability, syncope, and even sudden cardiac death (incidence: 4%) (2, 3). In hemodynamically stable patients, pharmacological conversion is generally used most for its efficacy. However, potential severe adverse events, such as transient asystole or hypotension, limit the range for treatment (2, 4, 5). Therefore, vagal maneuvers (VagMs) are still highly recommended for acute treatment in patients with regular SVT in current guidelines and the newest edition of advanced life support (6–8).

Vagal maneuvers are techniques aimed at increasing vagal parasympathetic tone and blocking the atrioventricular (AV) node. Carotid sinus massage (CSM) is a traditional maneuver performed by giving a firm pressure to the carotid sinus in the upward and downward directions then posteriorly and medially between the examiner's fingers and the patient's cervical vertebra for 5–10 s (9, 10). However, the potential risk of thromboembolic events should be a concern when deploying CSM in older patients. The standard Valsalva maneuver (SVM) may be currently the most used VagM because of safety and easy performance. The steps to perform SVM require patients to maintain a sitting position whilst keeping an expiratory pressure of around 30–40 mmHg by blowing into a syringe for 15 s. Regretfully, the conversion rate is generally < 20% because of inadequate blow time and pressure by persons (9, 11–14). The modified Valsalva maneuver (MVM) is thereby developed by placing the patient into a supine position with the leg raised promptly for 45 s following the steps of SVM (5). The diving reflex triggered by breath holding and cold water immersion may also stimulate vagal nuclei in the brain to ameliorate AV nodal conduction (15).

Several studies attempted to evaluate the efficacy and adverse events amongst different VagMs (5, 9, 10, 12, 16, 17). These studies seemly suggested MVM as the optimal VagM for SVT. However, small number of cases were included in these reports. On the basis of the principle of evidence-based appraisal, the comparison of effectiveness and adverse events amongst all VagMs remains unclear. Thus, we have performed a systemic review and a network meta-analysis (NMA) to investigate all VagMs in adult patients with SVT, identify the effectiveness of treatment, and report the potential adverse events.

## Materials and Methods

We performed a systematic review and NMA in accordance with the latest statement of the Preferred Reporting Items for Systematic Reviews and Meta-Analysis (PRISMA 2020) (18). We registered the systematic review protocol on the international website INPLASY with the registration number INPLASY2020110082 (DOI: 10.37766/inplasy2020.11.0082) and updated and recorded changes in protocol on June 20, 2021.

### Data Sources and Search Strategy

PubMed, Embase, Cochrane Library, Web of Science, and the Chinese National Knowledge Infrastructure (CNKI), were systematically searched by two independent investigators (CH Chen CH and CY Fan) from inception until August 10, 2021 to enroll adequate studies. We also searched the trial registry website clinicaltrials.gov, related congress proceedings, and references in relevant published articles. Keywords for search included “supraventricular tachycardia,” “室上性心動過速,” “室上速,” “Valsalva,” “maneuvers or manoeuvre,” “Valsalva 动作,” “carotid sinus massage,” “ice immersion,” “breath holding,” “vagal maneuver/manoeuvre,” and “迷走神經刺激術”. Our search strategy aimed to include every clinical trial investigating the use of non-invasive maneuvers in adult patients with SVT. One senior author (EPC Huang) supervised and confirmed the process of searching.

### Study Selection

Two authors (CH Chen and CY Fan) independently examined references using title and abstract. Full texts of relevant studies were retrieved. Studies published in languages other than English were also included after appropriate translation. We included randomized controlled trials (RCTs) involving adult patients (≥18 years old) with SVT and comparison of at least two VagMs for rhythm conversion before pharmacologic treatment. The exclusion criteria were as follows: (1) did not meet the inclusion criteria; (2) reviews, case reports or case series, letter to editors, commentaries or conference abstracts; and (3) incorrect study design. RCTs investigating patients with SVT undergoing electrophysiological study with multiple tests of VagMs were also excluded. One senior author (EPC Huang) supervised and confirmed the process of study selection.

### Data Extraction and Quality Assessment

Data were extracted from eligible studies included by two authors (CH Chen CH and CY Fan) individually, and the senior author (PC Lai and YT Huang) finalized the data. The data extracted from eligible studies included authors, publication year, study design, sex, age, numbers of cases and controls, initial heart rate, outcome, and adverse events. The risk of bias (RoB) and internal validity were assessed by two authors (PC Lai and YT Huang) independently by using the “Risk of bias assessment 2.0 (ROB 2.0) tool” developed by the Cochrane Collaboration (19). Divergences were resolved by consensus. RoB bar chart for the comparison of VagMs was depicted by the web-based “Confidence In Network Meta-Analysis” (CINeMA; https://cinema.ispm.unibe.ch), an online tool assessing confidence in the results of a NMA (20).

### Outcome Measures

The primary outcomes were the rate to convert SVT to sinus rhythm after VagMs, including initial and final response rates. The initial response rate represented the success rates immediately after the intervention. The secondary outcome was the risk of adverse events, including hypotension, nausea, dyspnea, arrhythmias, dizziness, or other patient discomfort mentioned by the authors.

### Data Synthesis, Statistical Analysis, and Sensitivity Analysis

We conducted NMAs on the basis of the frequentist approach with the random-effects model to compare the successful rate of conversion to sinus rhythm amongst different VagMs. The statistic investigation implemented through the “netgraph” and “netmeta” packages in the R software. Per comparison contribution matrix was also calculated in the CINeMA, which communicated to an R back-end server in the setting of random-effects model (21, 22). We presented the calculated estimates as relative risk (RR) with 95% CI. The order of probability in treatment effects and adverse events was ranked through the surface under the cumulative ranking curve analysis (SUCRA). A high SUCRA value in the endpoint of “conversion to sinus rhythm” and “adverse effects” indicated an effective and a safe maneuver, respectively. Publication bias was depicted by funnel plot and determined using the Egger test. *P*-value < 0.05 was considered as statistically significant. When conducting sparse networks, the sensitivity analysis was suggested from the Grading of Recommendations, Assessment, Development and Evaluation (GRADE) Working Group (23). Therefore, we further performed the fixed- and random-effects models by using the Bayesian and frequentist frameworks in the MetaInsight V3.14. website (https://crsu.shinyapps.io/MetaInsight/) based on the “gemtc,” “BUGSNET,” and “netmeta” packages in the R software (24). In frequentist and Bayesian NMAs, the ranges of prediction were presented as 95% confidence interval (CI) and 95% credible interval (CrI), respectively. Inconsistency between direct and indirect comparisons of different VagMs was also implemented through the MetaInsight V3.14. website. For rating the certainty of evidence (CoE) in the domains of inconsistency and publication bias, pairwise meta-analyses and Luis Furuya–Kanamori (LFK) index were proceeded through the Microsoft Excel (Microsoft, Redmont, WA, USA) add-in MetaXL 5.3 (EpiGear International, Sunrise Beach, Australia) by using the inverse variance heterogeneity model (25). The LFK indices outside the −1 and +1 interval were defined as publication bias (26).

### CoE Rating

The CoE for NMA was judged in accordance with the policy of the GRADE Working Group (27). The final CoE in each endpoint was rated as high, moderate, low, or very low. The online GRADEpro software (available from gradepro.org) was used to calculate the anticipated absolute effects based on odds ratio (OR).

## Results

### Study Characteristics

**Figure 1:**
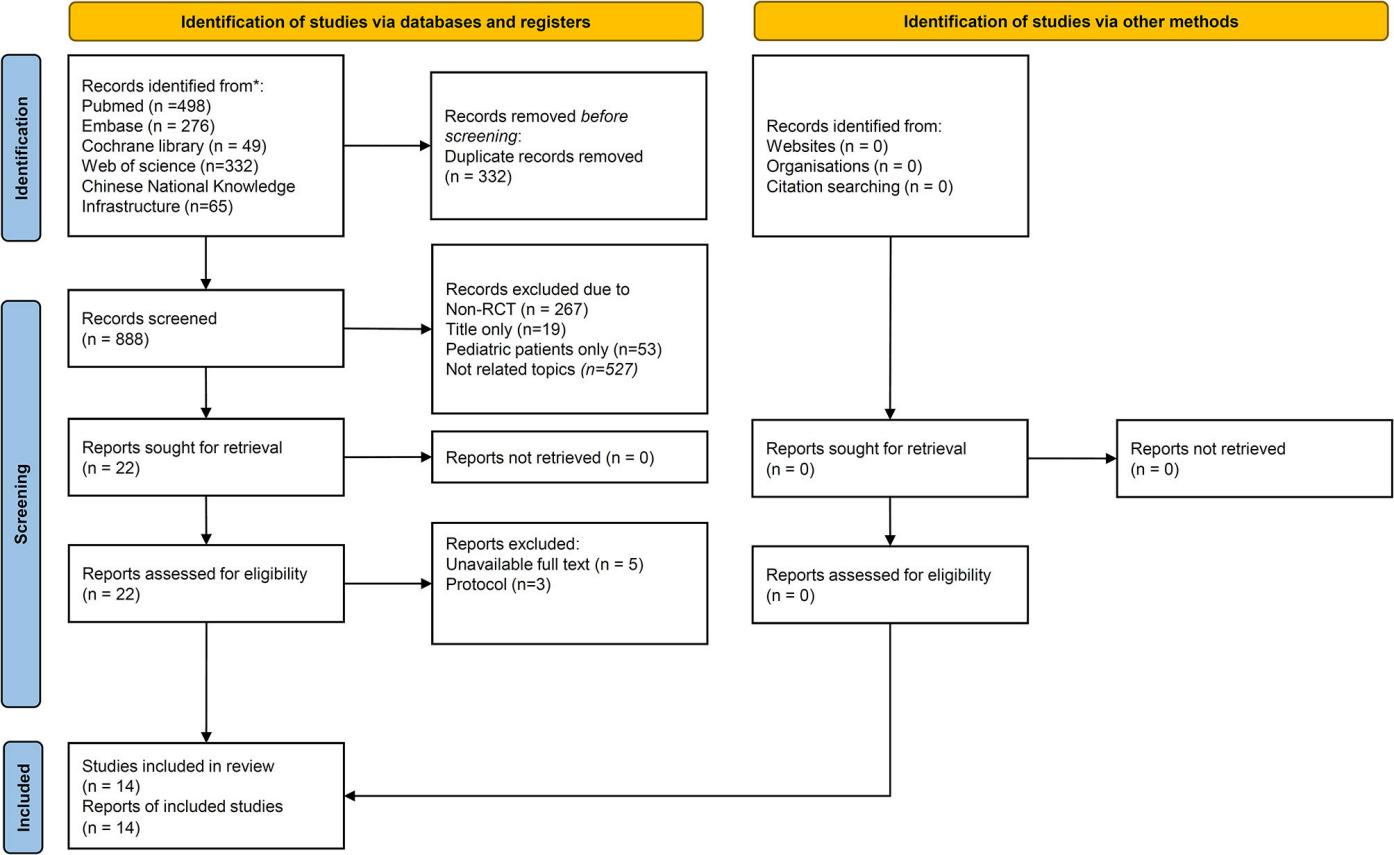
The screening process of the included studies.

The flow diagram of PRISMA is presented in Figure 1. A total of 1,220 studies were identified after completing the literature search. Amongst them, 332 papers were excluded due to duplication. After the screening of 888 papers, 866 were excluded due to the following: non-RCTs, focus on pediatric patients, case reports, title only, or not related topics. A total of 22 papers were screened for eligibility. Finally, 14 studies were enrolled for successful conversion to sinus rhythm, and 13 studies were analyzed to compare the risk of adverse events in different VagMs (5, 9, 10, 16, 28–35).

**Figure 2:**
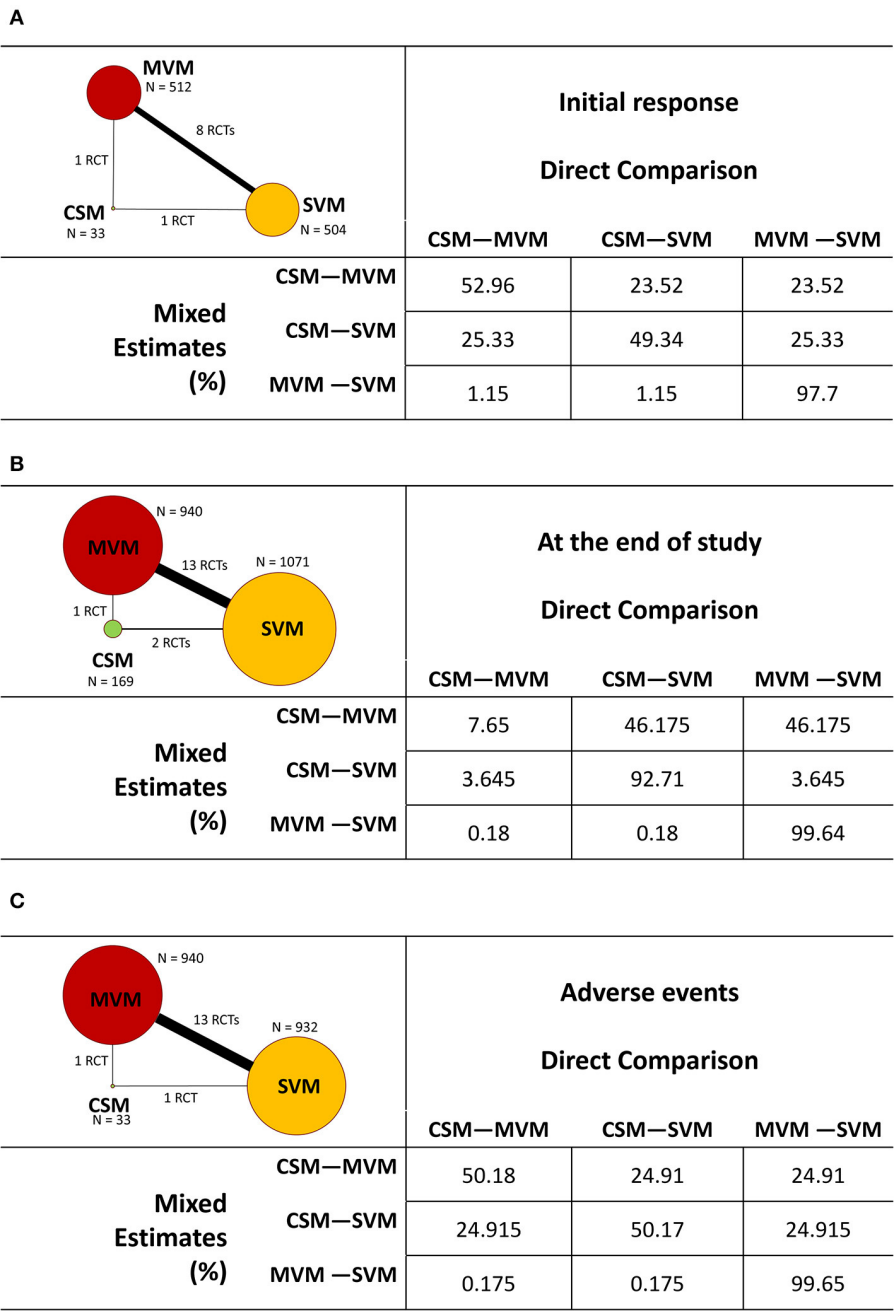
Mapping of the network diagram of vagal maneuvers and per comparison contribution matrix in returning to the sinus rhythm at **(A)** initial response, **(B)** end of study, and **(C)** adverse events. CSM, carotid sinus message; MVM, modified Valsalva maneuver; N, number; RCT, randomized controlled trial; SVM, standard Valsalva maneuver.

The characteristics of included studies are summarized in Table 1. A total of 2,180 patients mostly in ED were enrolled. Three different VagMs, including SVM, MVM, and CSM, were investigated in these studies, and only one RCT investigated all the abovementioned VagMs (10). Figure 2 not only depicts the mapping of the network diagram in each endpoint but also the contribution matrix presented as columns and rows corresponding to the percentage contribution of direct and network estimates, respectively. There was no indirect estimate contributed to the result of NMA because close loop network was observed in all endpoints. Because most of the RCTs compared the benefits and adverse events between MVM and SVM, naturally, the mixed estimates of the difference between MVM and SVM mainly came from direction comparison. Only one RCT reported the comparison between MVM and CSM; hence, the ratio of direction comparison to the mixed estimates of MVM–CSM was around half in the endpoints of initial response of converting to sinus rhythm (Figure 2A) and adverse events (Figure 2C). After calculation, the contribution percentage of MVM–CSM direction comparison in the endpoint of converting to sinus rhythm at the end of study was only 7.65% (Figure 2B).

**Table 1:** Basic characteristics of the studies included.

**Study**	**Design**	**Country**	**Setting**	**Group (*n*)**	**Age, years**	**Sex (M/F)**	**Heart rate, /min**	Conversion rate^*^, *n* (%)	**Adverse events, *n* (%)**
Lim et al. (9)	RCT	Singapore	ED	SVM = 139 CSM = 136	Total = 47.2 ± 18.3	Total = 63/85	N/A	SVM = 25 (17.9) CSM = 16 (11.7)	N/A
Appelboam et al. (5)	RCT	UK	ED	SVM = 214 MVM = 214	SVM = 54.5 ± 16.8 MVM = 55.1 ± 16.3	SVM = 80/134 MVM = 89/125	SVM = 179 ± 29 MVM = 172 ± 29	SVM = 37 (17) MVM = 93 (43)	SVM = 8 (4) MVM = 13 (6)
Li et al. (28)	RCT	China	ED & Admission	SVM = 80 MVM = 80	SVM = 52.0 ± 8.4 MVM = 54.0 ± 8.9	SVM = 56/24 MVM = 57/23	SVM = 173 ± 8 MVM = 176 ± 8	SVM = 24 (30) MVM = 62 (77.5)	SVM = 3 (3.7) MVM = 4 (5.0)
Çorbacioglu et al. (16)	RCT	Turkey	ED	SVM = 28 MVM = 28	SVM = 48 (20) MVM = 44 (20)	SVM = 13/15 MVM = 10/18	SVM = 180 (160–95) MVM = 180 (160–201)	SVM = 3 (10.7) MVM = 12 (42.9)	SVM = 2 (7.1) MVM = 2 (7.1)
Ceylan et al. (10)	RCT	Turkey	ED	SVM = 33 MVM = 32 CSM = 33	SVM = 61 (21) MVM = 50 (25) CSM = 63 (20)	SVM = 14/19 MVM = 17/15 CSM = 14/19	SVM = 167 (147–187) MVM = 177 (165–192) CSM = 168 (147–183)	SVM = 2 (6.1) MVM = 9 (28.1) CSM = 1 (3.0)	SVM = 0 (0) MVM = 0 (0) CSM = 0 (0)
Chen et al. (17)	RCT	China	ED	SVM = 119 MVM = 119	Range Total = 18–70	N/A	N/A	SVM = 19 (16) MVM = 55 (46)	SVM = 1 (0.8) MVM = 2 (1.6)
Huang et al. (30)	RCT	China	Admission	SVM = 34 MVM = 34	SVM = 53.2 ± 1.9 MVM = 56.0 ± 2.1	SVM = 16/18 MVM = 14/20	SVM = 178.67 ± 2.01 MVM = 180.83 ± 2.39	SVM = 10 (29.4) MVM = 21 (61.8)	SVM = 5 (11.8) MVM = 3 (8.8)
Gong et al. (29)	RCT	China	ED	SVM = 48 MVM = 48	SVM = 48.15 ± 8.35 MVM = 47.73 ± 9.81	SVM = 23/25 MVM = 28/20	SVM = 173.49 ± 9.57 MVM = 174.81 ± 8.66	SVM = 5 (10.4) MVM = 22 (45.8)	SVM = 4 (8.3) MVM = 2 (4.2)
Zhang et al. (36)	RCT	China	ED & Admission	SVM = 48 MVM = 50	SVM = 45.50 ± 10.24 MVM = 46.26 ± 12.02	SVM = 23/25 MVM = 20/30	SVM = 179.83 ± 14.39 MVM = 176.42 ± 14.54	SVM = 8 (16.7) MVM = 20 (40.0)	SVM = 3 (6.3) MVM = 3 (6.0)
Xiao et al. (34)	RCT	China	ED & Admission	SVM = 20 MVM = 20	SVM = 54.85 ± 9.73 MVM = 53.83 ± 9.61	SVM = 13/7 MVM = 11/9	N/A	SVM = 8 (40.0) MVM = 16 (80.0)	SVM = 6 (30.0) MVM = 6 (30.0)
Long et al. (31)	RCT	China	Admission	SVM = 33 MVM = 33	SVM = 55.1 ± 2.2 MVM = 58.0 ± 1.8	SVM = 14/19 MVM = 10/23	SVM = 177.88 ± 1.83 MVM = 183.94 ± 2.49	SVM = 9 (27.3) MVM = 17 (51.5)	SVM = 6 (30.0) MVM = 6 (30.0)
Song et al. (32)	RCT	China	ED	SVM = 63 MVM = 70	SVM = 56 ± 8 MVM = 55 ± 7	SVM = 31/32 MVM = 36/34	N/A	SVM = 9 (14.3) MVM = 9 (12.9)	SVM = 5 (7.9) MVM = 3 (4.3)
Wang et al. (33)	RCT	China	Admission	SVM = 181 MVM = 180	SVM = 49.29 ± 13.59 MVM = 51.76 ± 12.02	SVM = 74/107 MVM = 84/96	N/A	SVM = 36 (62.2) MVM = 112 (19.9)	SVM = 14 (7.7) MVM = 20 (11.1)
Wei et al. (35)	RCT	China	ED	SVM = 31 MVM = 32	SVM = 52.47 ± 3.30 MVM = 52.63 ± 3.42	SVM = 19/12 MVM = 18/14	N/A	SVM = 14 (45.2) MVM = 26 (81.3)	SVM = 5 (12.5) MVM = 4 (16.1)

*RCT, randomized controlled trial; ED, emergency department; CSM, carotid sinus massage; MVM, modified Valsalva maneuver; M/F, male/female; N/A, not available; SVM, standard Valsalva maneuver. Continuous variables were reported as median (interquartile rang) or mean ± standard deviation*

* *Successful rate of conversion to sinus rhythm at the endpoint of each study*.

### RoB Assessment

**Figure 3:**
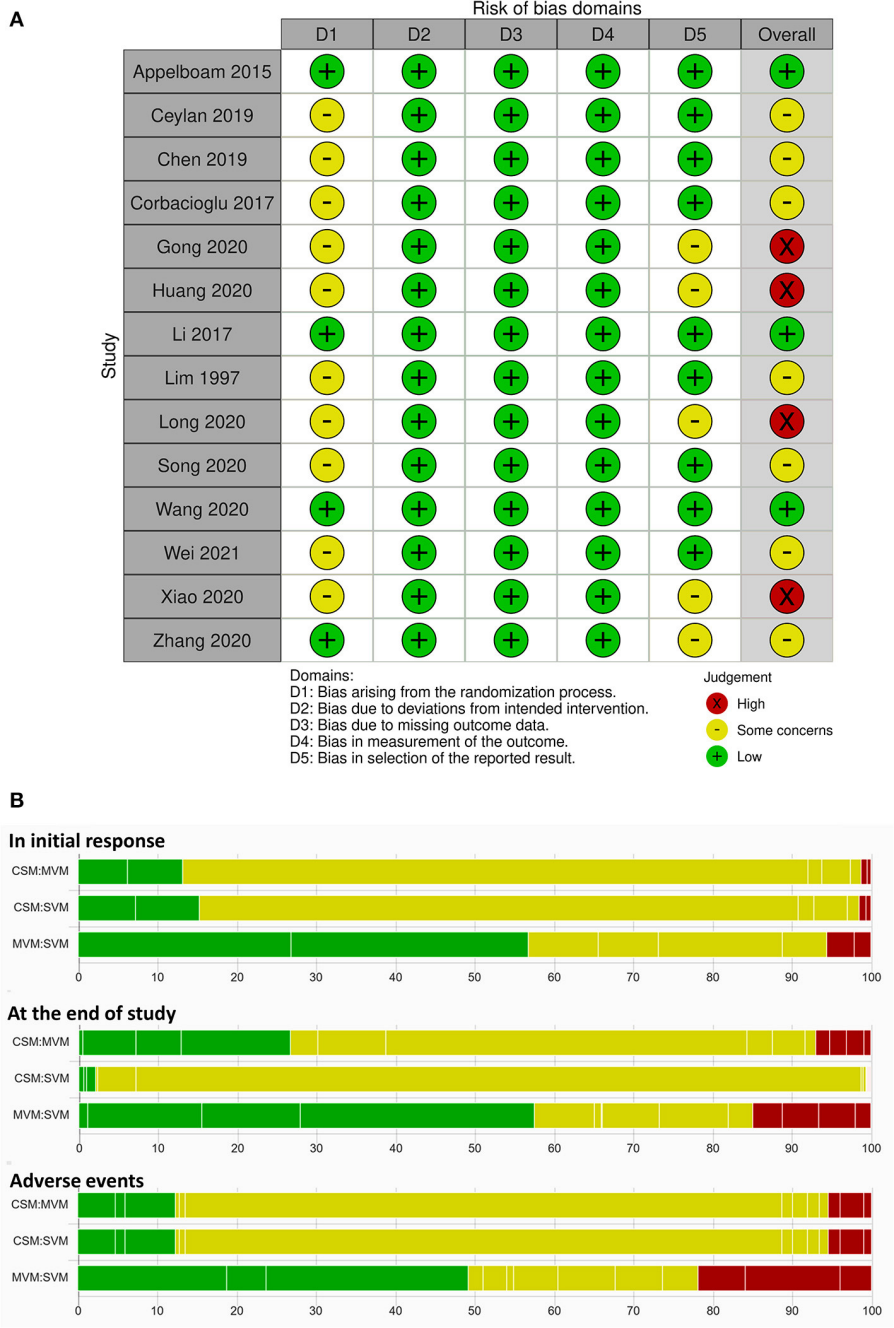
**(A)** Risk of bias in all included studies by using the revised Cochrane risk-of-bias tool. **(B)** Risk-of-bias bar chart for comparison of each vagal maneuver weighted by contribution matrix.

The assessment of RoB is summarized in Figure 3A. In the domain of randomization, all enrolled RCTS were judged as “some concern” due to no information about concealment. We judged one article (9) with “some concern” on the basis of the presence of gender and age differences of the control and intervention groups, which might cause baseline imbalance. Considering that SVT is a life-threatening condition and requires management immediately, pharmacological approach will be ordered within few periods if VagM failed. Performance bias might not be a critical issue amongst VagMs in RCTs in the consideration of blindness. In the domain of reporting bias, we judged six RCTs as “some concern” because of no prespecified plan, which would lead to selective reporting (29–31, 34, 36). In summary, the overall RoB results were “low,” “some concern,” and “high” in 3, 8, and 4 enrolled trials, respectively. Considering the overall RoB of each study in relative contribution to the mixed estimates, RoB bar charts of all endpoints are depicted in Figure 3B. Each bar represents the portion of RoB distribution of low (green), some-concern (yellow), and high (red) overall RoB based on the results of contribution matrix with white vertical lines, which separated the colored areas by to the contribution of each study. MVM–SVM estimates, the most frequent comparison in this NMA, were contributed from approximately half low overall RoB studies in all endpoints. In the endpoint of initial response, study-end response, and adverse events, percentage values of high overall RoB contributed to 5, 15, and 22% of MVM–SVM estimates from 2, 4, and 3 RCTs, respectively. Both estimates of CSM–MVM and CSM–SVM groups were generally contributed from some-concern overall RoB studies in all endpoints.

### Quantitative Data Synthesis

#### Conversion to Sinus Rhythm

**Figure 4:**
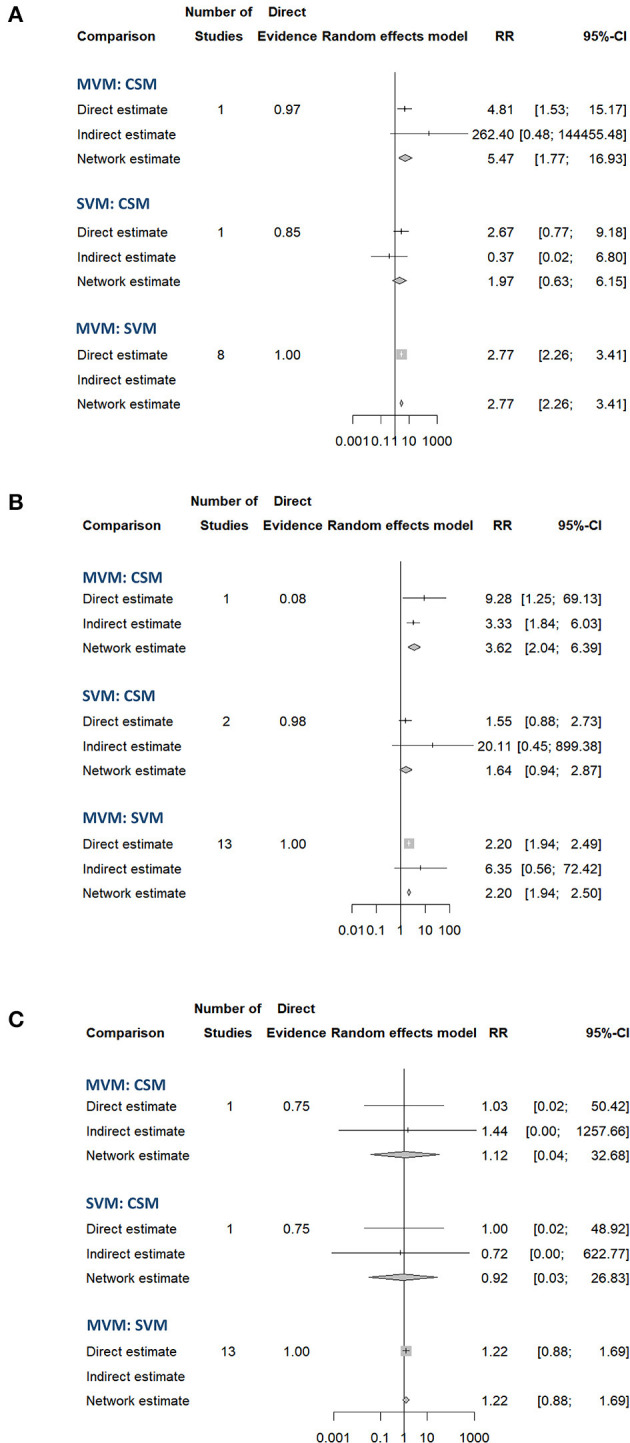
Forest plot of the pooled effect comparing the successful conversion rate to the sinus rhythm at **(A)** initial response, **(B)** end of study, and **(C)** adverse effects of different vagal maneuvers.

The result of NMA on the treatment effect of different VagMs was presented in the forest plot (Figure 4). In terms of initial response (eight RCTs), MVM yielded the best maneuver for successful conversion to sinus rhythm compared with SVM (RR: 2.77, 95% CI: 2.26–3.41) and CSM (RR: 5.47, 95% CI: 1.77–16.93; Figure 4A). At the end of each study, MVM was the most effective in converting SVT to sinus rhythm compared with SVM (RR: 2.20, 95% CI: 1.94–2.50) and CSM (RR: 3.62, 95% CI: 2.04–6.39; Figure 4C). Although SVM showed higher conversion rate than CSM, 95% CIs in both timepoints crossed the non-significance line of 1 in RR (Figures 4A,B). Inconsistency between direct and indirect comparisons amongst maneuvers in both endpoints yielded no statistical significance (Supplementary Table 1). Funnel plots depicted no statistical significance in terms of publication bias (Supplementary Figures 1A,B).

**Figure 5:**
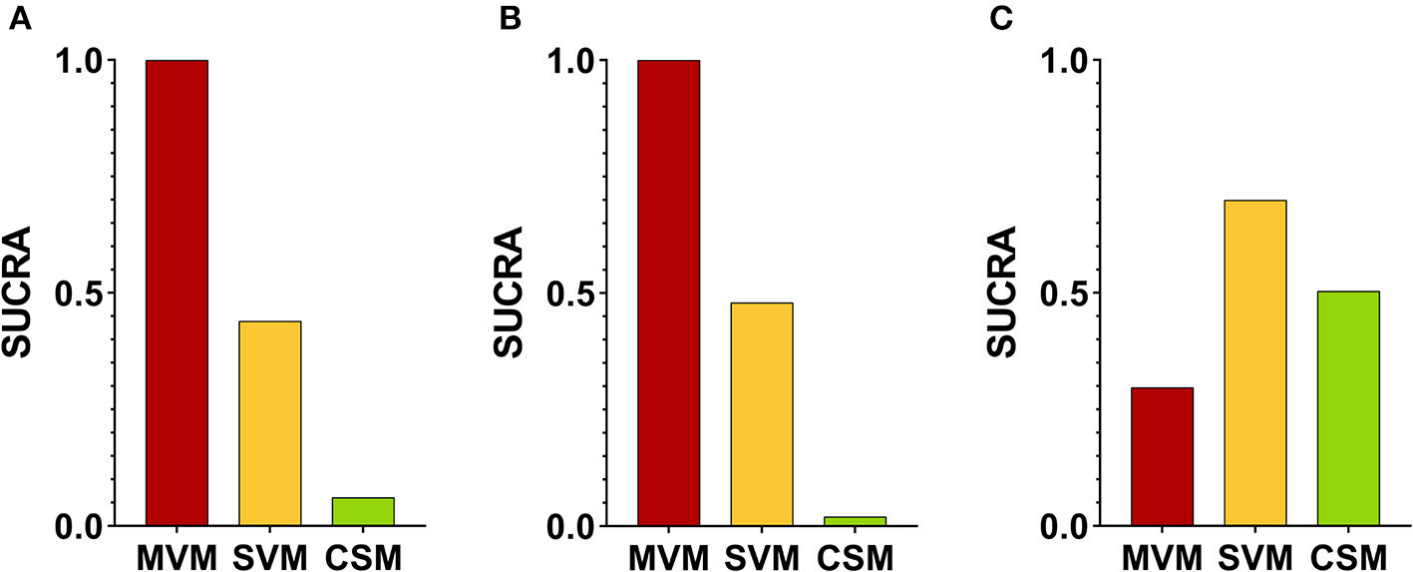
SUCRA values at **(A)** initial response, **(B)** end of study, and **(C)** adverse effects amongst different vagal maneuvers. SUCRA, surface under the cumulative ranking curve analysis.

The highest SUCRA values in the endpoint of initial response (Figure 5A) and the end of study (Figure 5B) in the MVM group were 0.9992 and 1.0000, respectively, which had overwhelmingly higher values than those in SVM and CSM groups. The second-highest SUCRA value amongst the three maneuvers was SVM in the initial response and at the end of the study (0.4395 and 0.4795, respectively). The SUCRA values of CSM at the initial and end-of-study responses (0.0613 and 0.0205, respectively) were lowest amongst the three VagMs. Compared with other VagMs, MVM was advanced in converting SVT to sinus rhythm. The sensitivity analysis did not show divergence when comparing fixed- and random-effects models of the Bayesian framework and fixed-effect frequentist approach (Supplementary Table 2). The ranges of intervals calculated by previously mentioned methodologies were also similar.

#### Adverse Events

Rare adverse events of VagMs were reported in these studies (Table 1). Most of the adverse events, including electrocardiography-captured events (asystolic pause and ventricular escape activity), hypotension, nausea, dyspnoea, and dizziness, were tolerable and spontaneously resolved after the cessation of the maneuver. Only one study reported the issue of adverse events in CSM, but no case was reported (10). The SUCRA values of MVM, SVM, and CSM were 0.2967, 0.6997, and 0.5036, respectively (Figure 5C). Although a higher risk of adverse events was estimated in the MVM group on the basis of the least SUCRA, all 94% CIs of RR between the comparison of two maneuvers crossed the non-significance line 1 of RR (Figure 4C). Inconsistency was not significant between the direct and indirect comparisons of maneuvers (Supplementary Table 1), and the funnel plot showed no statistical significance in terms of publication bias (Supplementary Figure 1C). The same trend was presented amongst the three models used in the sensitivity analysis of comparison between MVM and SVM, but 95% CrI or RR yielded above 1 (1.03–1.89) in the Bayesian approach with fixed-effect model (Supplementary Table 2). Regarding the adverse event comparison of MVM with CSM and CSM with SVM, extremely wide ranges of 95% CrIs crossing the line of 1 in RR and extremely high or low pooled estimates of RR were observed in the Bayesian approach with fixed- and random-effects models (Supplementary Table 2).

### Certainty of Evidence

We only chose two endpoints to rate the CoE, return to sinus rhythm at the end of study, and adverse events of VagMs because we considered both endpoints as most critical issues in this topic. On the basis of the protocol of NMA by the GRADE Working Group, direct and indirect comparisons should be considered in rating CoE (27). The parameters for CoE rating in pairwise comparison, including I^2^ for domain of inconsistency and LFK index for publication bias, are listed in Supplementary Table 3. The pooled estimates of CSM were set as reference. Detailed judgements of CoE in direct, indirect, and network comparisons are presented in Supplementary Table 4. We understood the difficulty of the randomization process in such emergent disorder, and we did not downgrade if “some concern” RoB was observed in the allocation bias. SVT is an acute illness with life-threatening potential, and the decision of treatment should be ordered in minutes. Thus, we believed that the allocation bias might not influence CoE. The CoE domains in direct rating in both endpoints were scored as high. Based on the annotation from the GRADE Working Group, the author should ignore the CoE from indirect comparison (27). In this study, we still presented the CoE domains in indirect rating as a reference document in Supplementary Table 4. To rate the indirect CoE involving the comparison between MVM and SVM, downgrading in one level was rated because more than 1/4 of RCTs were some-concern overall RoB in the reporting bias. Finally, the CoE of the network estimate regarding the best VagM of MVM in converting to sinus rhythm at the end of study was high (Supplementary Table 4; Table 2). The CoEs of adverse events in VagMs were low (Supplementary Table 4; Table 2). The rate of return to sinus rhythm at the end of the study after CSM was based on the crude estimate from the two enrolled RCTs, and the ratio of adverse events was set as 1% in accordance with a previous report on CSM (37). In comparison with that by CSM, a mean of 343 cases might be more successful to convert to sinus rhythm by MVM in every 1,000 patients with SVT.

**Table 2:** Certainty of evidence (CoE) in effects of vagal maneuvers.

**Estimates of effects, CIs, and CoE for patients with supraventricular tachycardia to return to sinus rhythm at the end of study by vagal maneuvers**					
**Patients:** SVT					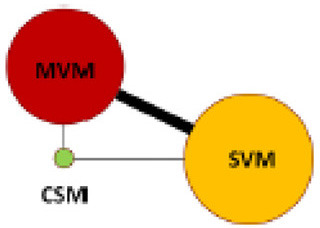
**Interventions:** MVM, SVM					
**Comparator (reference):** CSM					
**Outcome:** conversion to sinus rhythm at the end of study and adverse events					
**Setting:** emergency department or admission					
**Outcome**	**Effects and confidence in the estimate of effects**				**Comments**
	**MVM**		**SVM**		
**Return to sinus rhythm at the end of study**					
**CSM** **Comparator** 101/1000^a^ (10.1%)	**OR 7.12** (3.63 to 13.96) Network estimate	**343 more per 1000** (189 more to 510 more)	**OR 1.73** (0.91 to 3.30) Network estimate	**62 more per 1000** (8 fewer to 169 more)	MVM demonstrated the best vagal maneuver, far better than others.
	⊕⊕⊕⊕**High** Confidence in estimate		**⊕⊕⊕○Moderate** Confidence in estimate due to imprecision		
**Rank** 3 (SUCRA 0.0205)	** Rank** 1 (SUCRA 1.0000)		** Rank** 2 (SUCRA 0.4795)		
	Based on 940 participants (13 RCTs)		Based on 1,071 participants (14 RCTs)		
**Adverse events**					
**CSM** **Comparator** 10/1000^b^ (1%)	**OR 1.14** (0.04 to 35.05) Network estimate	**1 more per 1000** (10 fewer to 251 more)	**OR 0.91** (0.03 to 27.83) Network estimate	**1 fewer per 1000** (10 fewer to 209 more)	Insufficient evidences of difference in adverse events among each vagal maneuver.
	**⊕⊕○○Low** Confidence in estimate due to imprecision		**⊕⊕○○Low** Confidence in estimate due to imprecision		
**Rank** 2 (SUCRA 0.5036)	** Rank** 3 (SUCRA 0.2967)		** Rank** 1 (SUCRA 0.6997)		
	Based on 940 participants (13 RCTs)		Based on 932 participants (13 RCTs)		

*CI, confidence interval; CSM, carotid sinus massage; MVM, modified Valsalva maneuver; OR, odds ratio; SVM, standard Valsalva maneuver; RCT, randomized controlled trial; SUCRA, surface under the cumulative ranking curve analysis*.

a *The rate of return to sinus rhythm at the end of study after CSM is based on the crude estimate from the two enrolled RCTs*.

b *The ratio of adverse events referred to previous report of CSM (37)*.

## Discussions

Clinical physicians applied MVM, SVM, and CSM for the conversion of SVT to sinus rhythm, but the most effective VagM had not been well-discussed. The preference of MVM was recommended in the newest edition of adult advanced life support in European Resuscitation Council Guidelines (8). However, rigorous appraisal was not illustrated in detail. Ceylan et al. compared the three VagMs and found that MVM was superior to SVM and CSM in terminating SVT (10). The small number of cases in each group of VagM was the limitation of the present study. The systemic review with a meta-analysis of Abdulhamid et al. demonstrated that the sinus rhythm was achieved 2.5 times more by MVM compared with that by SVM (38). However, CSM was not included in that systematic review, and CoE was not rated. The benefit of NMA was that it could compare the results related to multiple interventions and provide mixed estimates in combination with direct and indirect information. Our presentation is the first study that used NMA to compare the pooled effect of different VagMs reported by previous RCTs. High-certainty evidence of pooling direct and indirect comparison presented that MVM was far superior to other VagMs in the successful conversion of SVT to sinus rhythm regardless of initial response and the end of studies in this closed-loop NMA. Although the pooled estimates of MVM vs. SVM were contributed from only half of the low RoB data, only small portion of high RoB data were correspondingly weighted in the results. Again, most of the RCTs appraised as some-concern overall RoB were due to the insufficient information in the domain of allocation, and we considered such condition may not elicit serious threaten in the CoE of the results. The online application CINeMA, which we used to demonstrate the contribution matrix and the following RoB bar charts, can also access the confidence of NMA designed by six domains of consideration: (i) within-study bias, (ii) reporting bias, (iii) indirectness, (iv) imprecision, (v) heterogeneity, and (vi) incoherence (21, 22). We favored to use the Cochrane RoB 2.0 tool and the CoE in GRADE because both methodologies have been widely recognized and applied in the field of evidence-based medicine for many years. Both provide detailed protocols based on comprehensive and rigorous considerations, and we believe the answers are objective if following the rules.

Vagal maneuvers were mostly protocolised through the use of manometer to ensure forced expiration reaching the standard pressure. Also, the response was studied at similar timepoints. Therefore, we assumed few heterogeneities and potential confounders amongst the included studies. In our NMA, with the comparison of MVM and CSM at the end of the study, the range of direct estimates was wider than that of indirect estimates in the frequentist approach with the random-effects model (Figure 4). This finding explained the importance of indirect comparison and the benefit of NMA. The GRADE Working Group recommended that the sensitivity analysis by using variant statistical methodologies, including Bayesian and frequentist frameworks, should be performed in sparse networks (23). The reason was that the marked widening ranges of the CIs/CrIs in indirect estimates had been observed in some NMA enrolling sparse studies compared with those in direct estimates (23). Such combining direct and indirect estimates results in marked widening of CIs/CrIs than direct estimates in some NMAs (23, 39, 40), and the benefit of NMA to increased precision (narrower CIs/CrIs compared with relying on direct estimates alone) is lost. The abovementioned condition may result from insufficient data in sparse networks to reliably estimate different variances across the network. Therefore, the GRADE Working Group recommended that the authors conducting an NMA of a sparse network should plan sensitivity analyses that lead to more trustworthy estimates of effects based on the optimal choices in statistical models (23). Failing to do so might result in network estimates with low CoE, making results less useful for patients, healthcare workers, and the guideline makers.

We paid more attention to be aware of the potential of incorrect analysis, especially when the direct comparison of MVM vs. CSM was based on only one RCT in our study. The uncertainty about the value of the between-study heterogeneity parameters in the random-effects model in the Bayesian framework might result in less precise estimates of treatment effects than that from frequentist frameworks in sparse NMA (41). The pooled estimates in our NMA worked in concert with this observation. Therefore, we chose the frequentist framework with the random-effects model as the primary statistical approach. As shown in Supplementary Table 2, consistent results with similar ranges of CIs/CrIs amongst all VagM comparisons were observed in the outcomes of converting to sinus rhythm at the initial response and the end of study with various statistical methodologies. Therefore, the judgement of decision might not change when choosing the pooled estimates from different statistics. Regarding the pooled estimates of adverse events, extremely wide ranges of 95% CrIs were observed in the Bayesian approach. This finding might result from some zero event in the study arm. To handle the event of zero case in the frequentist approach, an arbitrary number of 0.5 was added to the event by the software. Such a correction resulted in narrow ranges of 95% CIs than that of 95% CrIs. Based on the CoE in GRADE, more RCTs are warranted to determine the evidence of adverse events amongst VagMs.

Our NMA demonstrated a conspicuous conversion rate of MVM compared with that of SVM. A reasonable explanation was that by adding a passive leg raise or an abrupt change from the semirecumbent position to the supine position after SVM, the modified version could increase venous return and maximize the vagal tone, leading to a decrease in heart rate by baroreflex and suppression of the AV node (17, 42, 43). This effect was instant and could sustain and maintain the successful conversion to sinus rhythm according to our result. In previous studies, the successful conversion rate from SVT to the sinus rhythm of MVM could reach ~50% (5, 16, 17). Nearly half of the patients with SVT could be successfully treated through a non-pharmacologic, free-of-charge, and simple maneuver. Although previous studies failed to show any reduction in the time of hospital stay or need of admission (5, 17), refraining from pharmacotherapy was more cost-effective and could prevent from potential severe adverse events of medications. Our result showed a potentially increased risk of adverse events in MVM compared with that in SVM. Most of the adverse events were acceptable and self-limited after the cessation of the maneuver. Thus, the adverse events might not be critical issues in performing MVM. Although no significantly increased adverse event was noted in the CSM group, some life-threatening complications, such as transient ischemic attack and stroke, had been put forward previously, especially in elderly patients (37, 44–48).

A simple handheld Valsalva assist device (VAD) had been developed to standardize the protocol of VagM reliably (49). In a recent RCT in pre-hospital settings, a higher conversion rate by VagM with VAD than usual strain method was observed (50). However, MVM was not routinely performed in both groups. Thus, the effectiveness of VAD should be further investigated. Aside from VagMs in this NMA, many ways could achieve an increasing vagal tone. These ways, which were not compared in the present study, included provoking a human diving reflex by ice packing, water immersion, or breath holding (51, 52). These procedures were seldom in clinical practice, and only some case series were reported especially for children (53). The use of a pneumatic antishock garment could also increase venous return and vagal tone (12). A new VagM, named reverse Valsalva maneuver, had been reported this year (54). RCTs are still the gold standard in validating the evidence of these VagMs. Literature regarding CSM is limited in this NMA. SVT induced by electrical stimulation involving CSM management was not included (55). Despite these limitations, MVM is the best VagM for patients with SVT due to its effectiveness with high CoE appraised by the modern methodology of GRADE.

## Conclusions

Based on the results of NMA and CoE appraisal by GRADE, MVM is the most optimal VagM due to its high conversion rate with no significant increase in adverse events. Therefore, we recommend the use of MVM, which is a reasonable, considerable, and practicable approach clinically and can be proceeded before the pharmacologic treatment of SVT.

## Data Availability Statement

The original contributions presented in the study are included in the article/Supplementary Material, further inquiries can be directed to the corresponding author/s.

## Author Contributions

EH and C-HC: database search, data extraction, critical analysis, interpretation of data, and drafting of the manuscript. C-YF: database search, data extraction, and drafting of the manuscript. C-WS: interpretation of data and revising the manuscript. PL and YH: double confirming of enrolled studies and data, concept of the network meta-analysis, statistical analyses, grading of risk of bias, and drafting of the manuscript. All authors contributed to the article and approved the submitted version.

## Conflict of Interest

The authors declare that the research was conducted in the absence of any commercial or financial relationships that could be construed as a potential conflict of interest. The reviewer Y-tK declared a shared affiliation, with the authors, EH, C-HC, C-YF, C-WS, to the handling editor at the time of the review.

## Publisher's Note

All claims expressed in this article are solely those of the authors and do not necessarily represent those of their affiliated organizations, or those of the publisher, the editors and the reviewers. Any product that may be evaluated in this article, or claim that may be made by its manufacturer, is not guaranteed or endorsed by the publisher.
