# Supplementary material for: Comparison of Various Vagal Maneuvers for Supraventricular Tachycardia by Network Meta-Analysis
Source: Front Med (Lausanne). 2022 Feb 3;8:769437. doi: 10.3389/fmed.2021.769437 (PMC8850969; doi:10.3389/fmed.2021.769437)
Supplement: Supplementary file 1 [file Data_Sheet_1.docx]

**Supplemental Table 1.** Analysis of inconsistency between direct and indirect comparison

| **Endpoint** | **Comparison** | **Q Statistics**  **Network** | **Q Statistics**  **Direct** | **Q Statistics**  **Indirect** | ***p* value** |
| --- | --- | --- | --- | --- | --- |
| **Return to sinus rhythm at initial response** | **CSM**  **vs**  **MVM** | -1.70 | -1.57 | -5.57 | 0.22 |
|  | **CSM**  **vs**  **SVM** | -0.68 | -0.98 | 0.99 | 0.22 |
|  | **MVM**  **vs**  **SVM** | 1.02 | 1.02 | N/A | N/A |
| **Return to sinus rhythm at the end of study** | **CSM**  **vs**  **MVM** | -1.29 | -2.23 | -1.20 | 0.34 |
|  | **CSM**  **vs**  **SVM** | -0.50 | -0.44 | -3.00 | 0.19 |
|  | **MVM**  **vs**  **SVM** | 0.79 | 0.79 | 1.85 | 0.39 |
| **Adverse events of each maneuver** | **CSM**  **vs**  **MVM** | -0.11 | -0.03 | -0.37 | 0.93 |
|  | **CSM**  **vs**  **SVM** | 0.08 | 0.00 | 0.33 | 0.93 |
|  | **MVM**  **vs**  **SVM** | 0.20 | 0.20 | N/A | N/A |

CSM, carotid sinus massage; MVM, modified Valsalva maneuver; SVM, standard Valsalva maneuver; N/A, not applicable.

**Supplemental Figure 1.** Funnel plots in return to sinus rhythm at (A) initial response, (B) end of study and (C) adverse events in random-effect model with Egger test.


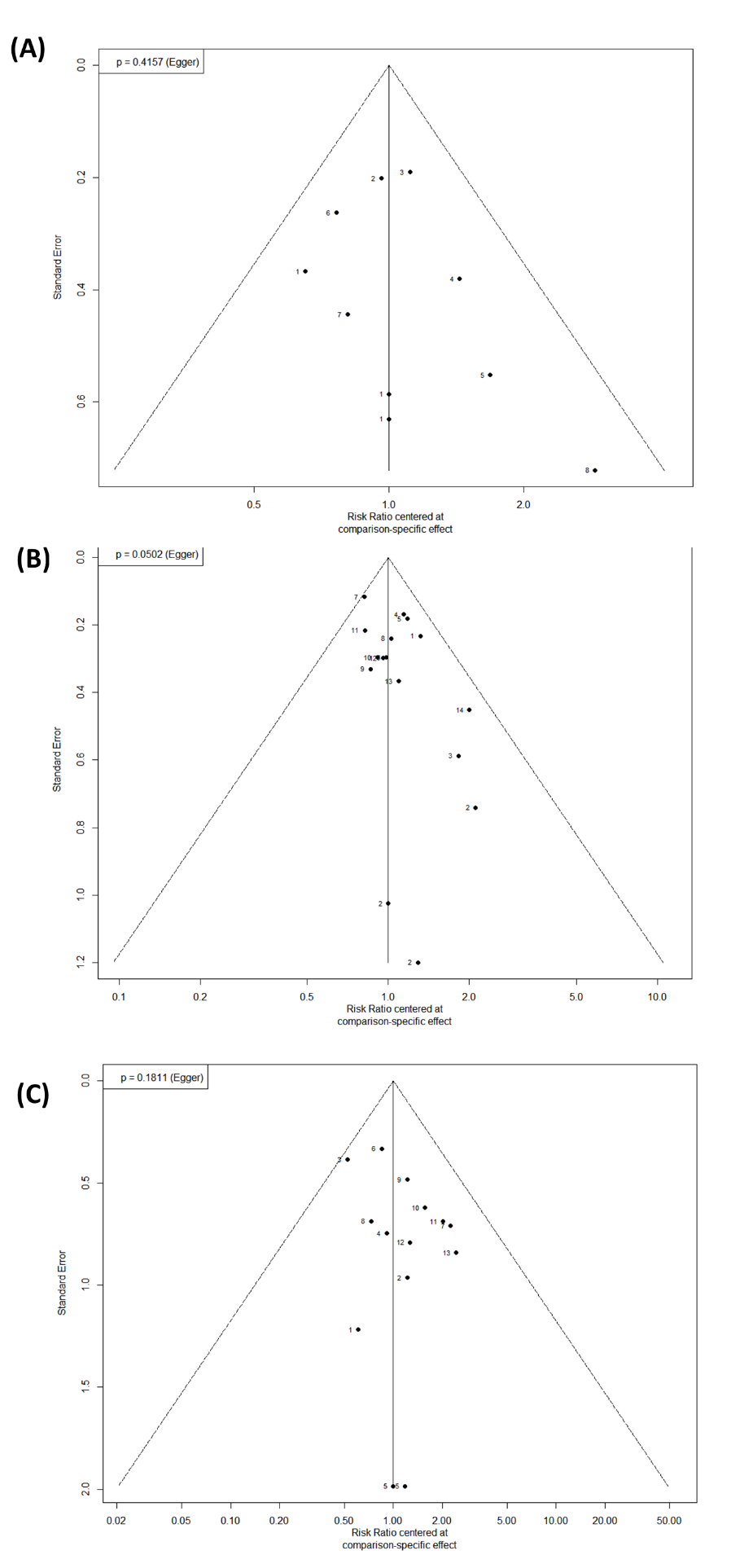


**Supplemental Table 2.** Sensitivity analysis using different models

| **Endpoint** | **Comparison** | **Bayesian**  **Random**  **(RR; 95%CrI)** | **Bayesian**  **Fixed**  **(RR; 95%CrI)** | **Frequentist**  **Fixed (RR; 95%CI)** |
| --- | --- | --- | --- | --- |
| **Return to sinus rhythm at initial response** | **MVM vs SVM** | **2.96 (2.20 – 4.23)** | **2.90**  **(2.38 – 3.60)** | **2.77 (2.26 – 3.41)** |
|  | **MVM vs CSM** | **4.65 (1.64 – 16.03)** | **4.30**  **(1.82 – 14.50)** | **5.47 (1.77 – 16.93)** |
|  | **CSM vs SVM** | 0.63  (0.18 – 1.88) | 0.68  (0.20 – 1.62) | 0.51 (0.16 – 1.58) |
| **Return to sinus rhythm at the end of study** | **MVM vs SVM** | **2.32 (1.98 – 2.81)** | **2.26 (1.99 – 2.58)** | **2.20 (1.94 – 2.50)** |
|  | **MVM vs CSM** | **3.50 (1.99 – 6.64)** | **3.43 (2.03 – 6.00)** | **3.62 (2.04 – 6.39)** |
|  | **CSM vs SVM** | 0.66 (0.35 – 1.23) | 0.66 (0.38 – 1.09) | 0.61 (0.35 – 1.07) |
| **Adverse events of each Valsalva maneuver** | **MVM vs SVM** | 1.37  (0.94 –1.99) | **1.40**  **(1.03 –1.91)** | 1.22 (0.88 – 1.69) |
|  | **MVM vs CSM** | 7.6×10^-3^  (3.99×10^-7^ – 1.16) | 3.3×10^-3^  (9.34×10^-9^ – 1.94) | 1.12 (0.04 – 32.68) |
|  | **CSM vs SVM** | 390.0  (0.27 –1.29×10^11^) | 122.0  (0.29 –1.07×10^7^) | 1.09 (0.04 – 31.69) |

CI, confidence interval; CrI, credible interval; CSM, carotid sinus massage; MVM, modified Valsalva maneuver, RR, relative risk; SVM, standard Valsalva maneuver.

**Supplemental Table 3. Pairwise meta-analysis by direct comparison in inverse variance heterogeneity model**

| **Endpoint** | **Comparison** | | **RR**  **(95% CI)** | **I^2^** | **LFK index** |
| --- | --- | --- | --- | --- | --- |
| **Return to sinus rhythm at the end of study** | **CSM**  **vs**  **MVM** | N = 1 | 9.28 (1.25 – 69.13) | N/A^1^ | N/A^2^ |
|  | **SVM**  **vs**  **CSM** | N = 2 | 1.55 (0.88 **–** 2.73) | 0% | N/A^2^ |
|  | **MVM**  **vs**  **SVM** | N = 13 | 2.20 (1.94 **–** 2.49) | 0% | 3.5 |
| **Adverse events of each maneuver** | **MVM**  **vs**  **CSM** | N = 1 | 1.03 (0.02 – 50.42) | N/A^1^ | N/A^2^ |
|  | **SVM**  **vs**  **CSM** | N = 1 | 1.00 (0.02 – 48.96) | N/A^1^ | N/A^2^ |
|  | **MVM**  **vs**  **SVM** | N = 13 | 1.22 (0.88 **–** 1.69) | 0% | -2.1 |

^1^ I-sqaure cannot be calculated when enrollment of only one study.

^2^ LFK index cannot be calculated when enrollment of only one or two studies.

CI, confidence interval; CSM, carotid sinus massage; MVM, modified Valsalva maneuver; RR, relative risk; SVM, standard Valsalva maneuver; LFK, Luis Furuya-Kanamori; N/A, not applicable.
