# Supplementary material for: Comparison of Various Vagal Maneuvers for Supraventricular Tachycardia by Network Meta-Analysis
Source: Front Med (Lausanne). 2022 Feb 3;8:769437. doi: 10.3389/fmed.2021.769437 (PMC8850969; doi:10.3389/fmed.2021.769437)
Supplement: Supplementary file 2 [file Data_Sheet_2.docx]

**Supplemental Table 4: Detailed ratings of domains in direct, indirect, and network meta-analysis**

| **Outcome** | **Comparison** | **Domains in direct rating** | **Domains in indirect rating** | | | **Network rating** |
| --- | --- | --- | --- | --- | --- | --- |
| **Return to sinus rhythm at the end of study** | **MVM vs CSM** | - RoB: not serious  - Inconsistency: N/A^1^  - Indirectness: not serious  - Publication bias: N/A^1^ | **MVM vs SVM** | **SVM vs CSM** | | - Highest rating between direct and indirect ratings: high  - Incoherence: not serious  - Imprecision: not serious |
|  |  |  | - RoB: serious^2^  - Inconsistency: not serious  - Indirectness: not serious  - Publication bias: serious^4^ | - RoB: not serious  - Inconsistency: not serious  - Indirectness: not serious  - Publication bias: N/A^3^ | |  |
|  |  |  | Intransitivity: not serious | | |  |
|  |  | Rating: high | Lowest of the rating: low | | | **Overall CoE: high** |
|  | **SVM vs CSM** | - RoB: not serious  - Inconsistency: not serious  - Indirectness: not serious  - Publication bias: N/A^3^ | **SVM vs MVM** | **MVM vs CSM** | | - Highest rating between direct and indirect ratings: high  - Incoherence: not serious  - Imprecision: serious^4^ |
|  |  |  | - RoB: serious^2^  - Inconsistency: not serious  - Indirectness: not serious  - Publication bias: serious^4^ | - RoB: not serious  - Inconsistency: N/A^1^  - Indirectness: not serious  - Publication bias: N/A^1^ | |  |
|  |  |  | Intransitivity: not serious | | |  |
|  |  | Rating: high | Lowest of the rating: low | | | **Overall CoE: moderate** |
| **Adverse events** | **MVM vs CSM** | - RoB: not serious  - Inconsistency: N/A^1^  - Indirectness: not serious  - Publication bias: N/A^1^ | **MVM vs SVM** | | **SVM vs CSM** | - Highest rating between direct and indirect ratings: high  - Incoherence: not serious  - Imprecision: very serious^5^ |
|  |  |  | - RoB: serious^2^  - Inconsistency: not serious  - Indirectness: not serious  - Publication bias: serious^4^ | | - RoB: not serious  - Inconsistency: N/A^1^  - Indirectness: not serious  - Publication bias: N/A^1^ |  |
|  |  |  | Intransitivity: not serious | | |  |
|  |  | Rating: high | Lowest of the rating: low | | | **Overall CoE: low** |
|  | **SVM vs CSM** | - RoB: not serious  - Inconsistency: N/A^1^  - Indirectness: not serious  - Publication bias: N/A^1^ | **SVM vs MVM** | **MVM vs CSM** | | - Highest rating between direct and indirect ratings: high  - Incoherence: not serious  - Imprecision: very serious^6^ |
|  |  |  | - RoB: serious^2^  - Inconsistency: not serious  - Indirectness: not serious  - Publication bias: serious^4^ | - RoB: not serious  - Inconsistency: N/A^1^  - Indirectness: not serious  - Publication bias: N/A^1^ | |  |
|  |  |  | Intransitivity: not serious | | |  |
|  |  | Rating: high | Lowest of the rating: low | | | **Overall CoE: low** |

CSM, carotid sinus massage; MVM, modified Valsalva maneuver; SVM, standard Valsalva maneuver; N/A, not applicable; RoB, risk of bias; CoE, certainty of evidence.

^1^ Only 1 study.

^2^ More than 1/4 of studies were some-concern RoB in reporting bias.

^3^ Only 2 studies.

^4^ Luis Furuya-Kanamori indexes >1 or < -1

^5^ Cross over the non-significant line in the range of 95% CI.

^6^ Very wide 95% confidence interval and cross over the non-significant line in the range of 95% confidence interval.
